# Nematode Predation and Competitive Interactions Affect Microbe-Mediated Phosphorus Dynamics

**DOI:** 10.1128/mbio.03293-21

**Published:** 2022-04-14

**Authors:** Jie Zheng, Francisco Dini-Andreote, Lu Luan, Stefan Geisen, Jingrong Xue, Huixin Li, Bo Sun, Yuji Jiang

**Affiliations:** a State Key Laboratory of Soil and Sustainable Agriculture, Institute of Soil Science, Chinese Academy of Sciences, Nanjing, China; b Department of Plant Science, The Pennsylvania State University, University Park, Pennsylvania, USA; c Huck Institutes of the Life Sciences, The Pennsylvania State University, University Park, Pennsylvania, USA; d Laboratory of Nematology, Wageningen University, Wageningen, Netherlands; e College of Resources and Environmental Sciences, Nanjing Agricultural University, Nanjing, China; Harvard University

**Keywords:** ALP-producing bacteria, ALP activity, competition, keystone taxa, nematode predation, phosphorus availability

## Abstract

Nematode predation plays an essential role in determining changes in the rhizosphere microbiome. These changes affect the local nutrient balance and cycling of essential nutrients by selectively structuring interactions across functional taxa in the system. Currently, it is largely unknown to what extent nematode predation induces shifts in the microbiome associated with different rates of soil phosphorous (P) mineralization. Here, we performed an 7-year field experiment to investigate the importance of nematode predation influencing P availability and cycling. These were tracked via the changes in the alkaline phosphomonoesterase (ALP)-producing bacterial community and ALP activity in the rhizosphere of rapeseed. Here, we found that the nematode addition led to high predation pressure and thereby caused shifts in the abundance and composition of the ALP-producing bacterial community. Further analyses based on cooccurrence networks and metabolomics consistently showed that nematode addition induced competitive interactions between potentially keystone ALP-producing bacteria and other members within the community. Structural equation modeling revealed that the outcome of this competition induced by stronger predation pressure of nematodes was significantly associated with higher diversity of ALP-producing bacteria, thereby enhancing ALP activity and P availability. Taken together, our results provide evidence for the importance of predator-prey and competitive interactions in soil biology and their direct influences on nutrient cycling dynamics.

## INTRODUCTION

The rhizosphere is a local interface that supports the exchange of resources between plants and the associated microbiome. Microorganisms colonizing the rhizosphere are integral to soil phosphorus (P) cycling and play essential roles in influencing plant growth, health, and performance (1). Understanding how specific microbial taxa contribute to plant P acquisition in the rhizosphere and properly manipulating these microorganisms to enhance P availability and uptake have been considered fundamental topics of research in the past few decades. Specific taxa within the soil microbiome are able to convert organic compounds into plant-available P via soil phosphatase hydrolysis (2). The bacterial alkaline phosphomonoesterases (ALPs) are the most thoroughly investigated in terms of their biosynthesis, catalytic properties, and genetic underpinning mechanisms (3). As part of the phosphate starvation (Pho) regulatory mechanism, the *phoD* gene was identified as coding for the production of ALP, and available databases have been using the *phoD* gene as a marker for the ALP gene (4, 5). The ALP production by bacteria has been demonstrated to be induced under conditions of low available inorganic P (6), and studies have shown the composition and abundance of the bacterial *phoD* to dynamically change due to manure and mineral P fertilization management (7, 8).

Biological interactions between the soil microfauna and microbial populations in the rhizosphere can determine rates of phosphorus cycling and affect the nutritional status of plants (9). For example, bacterial predation by nematodes has been shown to influence the decomposition and nutrient release rates in the rhizosphere (10, 11). Since distinct bacterial taxa are not equally susceptible to predation by nematodes, the presence of nematodes often imposes selective changes in the resident microbiome with consequences for ecological interactions (12). In this scenario, the potential keystone taxa within the microbiome community often modulate the diversity and patterns of species interactions, which collectively relate to patterns of community structure and other ecological properties, such as community resilience and resistance (13, 14). Mounting theoretical and empirical evidence supports that soil microbial diversity is largely supported by dynamic interspecific interactions, including competition and predation (15, 16). However, we still lack knowledge of how and the extent to which nematode selective predation on bacterial taxa affects fundamental aspects of soil biology.

Recent studies have been using the quantification of *phoD* gene abundance to infer potentially positive correlations between ALP-producing bacteria with ALP activity affecting P availability across divergent systems (5, 17). Most importantly, drawing inferences on diversity-functioning relationships often relies on comprehensively understanding the ecology of interactions and the physiology of individual taxa. For example, it has been shown that competition with the genus *Arthrobacter* (termed the bacterial “keystone”) and with the genus *Chaetomium* (termed the fungal “keystone”) significantly stimulated bacterial and fungal diversity, respectively, and resulted in a reduction of carbohydrate catabolism in the system (14). In fact, there remains a paucity of studies investigating keystone taxa, their functional importance, and their changes due to biological interactions with other members within the community (18, 19). As such, exploring the dynamic shifts in the microbial community mediated by the selective predation by nematodes and ecological competition between taxa might help to advance our understanding of soil nutrient cycling dynamics mediated by ecological interactions.

Here, we aimed to mechanistically understand how selective predation affected bacterial keystone taxa resulting in the modulation of ALP-producing bacterial community and ALP activity in the rhizosphere. We used a 7-year field site treated with different fertilizers annually to test the biological and chemical implications of nematode addition. Soil samples were taken from the rhizosphere of rapeseed in a potato-rape crop rotation system. We aimed at answering the following questions. (*i*) Does nematode addition alter the abundance and composition of the ALP-producing bacterial community in the rhizosphere? (*ii*) Does nematode addition increase the diversity of ALP-producing bacteria by affecting keystone taxa in the community? And, if so, (*iii*) how does ALP activity respond to nematode addition via changes in the rhizosphere ALP-producing bacterial community?

## RESULTS

### Soil physicochemical properties and enzymatic activities.

One-way analysis of variance (ANOVA) indicated that fertilization treatments significantly affected the rhizosphere soil properties, including soil organic carbon (SOC), total nitrogen (TN), alkali-hydrolyzable nitrogen (AN), available phosphorus (AP), and available potassium (AK) (analysis of variance *F* test, *F*_(7, 16)_ = 2.99 to 20.34, *P *< 0.01) but not pH (*P *= 0.926), total phosphorus (TP; *P *= 0.092), and total potassium (TK; *P *= 0.940) (see Table S1 in the supplemental material). Compared to treatments without nematode addition (FO, chemical fertilizer with organic manure; FOE, FO plus earthworms; FOP, FO plus phosphate-solubilizing bacterium; and FOPE, FO plus phosphate-solubilizing bacterium and earthworms), the contents of SOC, TN, AN, and AP with nematode addition (FON, FO plus nematodes; FOEN, FO plus nematodes and earthworms; FOPN, FO plus phosphate-solubilizing bacterium and nematodes; and FOPEN, FO plus phosphate-solubilizing bacterium, earthworms, and nematodes) increased by 5.3%, 9.5%, 12.4%, and 11.5%, respectively (Table S1) (*P *< 0.05). There were significant differences in soil phosphomonoesterase activities across the fertilization treatments (*F*_(7, 16)_ = 2.42 to 20.11, *P *< 0.05). The acid and alkaline phosphomonoesterase (ACP and ALP) activities peaked under the FOPEN treatment and displayed the lowest value under the FO treatment (Fig. S1). In particular, ACP and ALP activities increased by 35.6% and 18.3%, respectively, with nematode addition relative to treatments without nematodes. AP was significantly correlated with ALP activity (*r *= 0.615, *P *= 0.001) but not with ACP activity (*r *= 0.204, *P *= 0.339).

10.1128/mbio.03293-21.1 FIG S1 Soil acid phosphomonoesterase (ACP; a) and alkaline phosphomonoesterase (ALP; b) activities in the rhizosphere. Bars with different letters are significantly different (*P *< 0.05) by Tukey’s HSD test. ACP and ALP activities showed significant differences between treatments with and without nematode addition. FO, chemical fertilizer with organic manure; FOE, FO plus earthworms; FOP, FO plus phosphate-solubilizing bacteria; FON, FO plus nematodes; FOPE, FO plus phosphate-solubilizing bacteria and earthworms; FOPN, FO plus phosphate-solubilizing bacteria and nematodes; FOEN, FO plus nematodes and earthworms; FOPEN, FO plus phosphate-solubilizing bacteria, earthworms, and nematodes. **, *P *< 0.01. Download FIG S1, TIF file, 0.5 MB. Copyright © 2022 Zheng et al. 2022 Zheng et al. https://creativecommons.org/licenses/by/4.0/ This content is distributed under the terms of the Creative Commons Attribution 4.0 International license.

10.1128/mbio.03293-21.6 TABLE S1 Rhizosphere soil physicochemical properties under eight fertilization treatments. Download Table S1, DOCX file, 0.02 MB. Copyright © 2022 Zheng et al. 2022 Zheng et al. https://creativecommons.org/licenses/by/4.0/ This content is distributed under the terms of the Creative Commons Attribution 4.0 International license.

### Influence of nematode addition on ALP-producing bacterial community.

**FIG 1:**
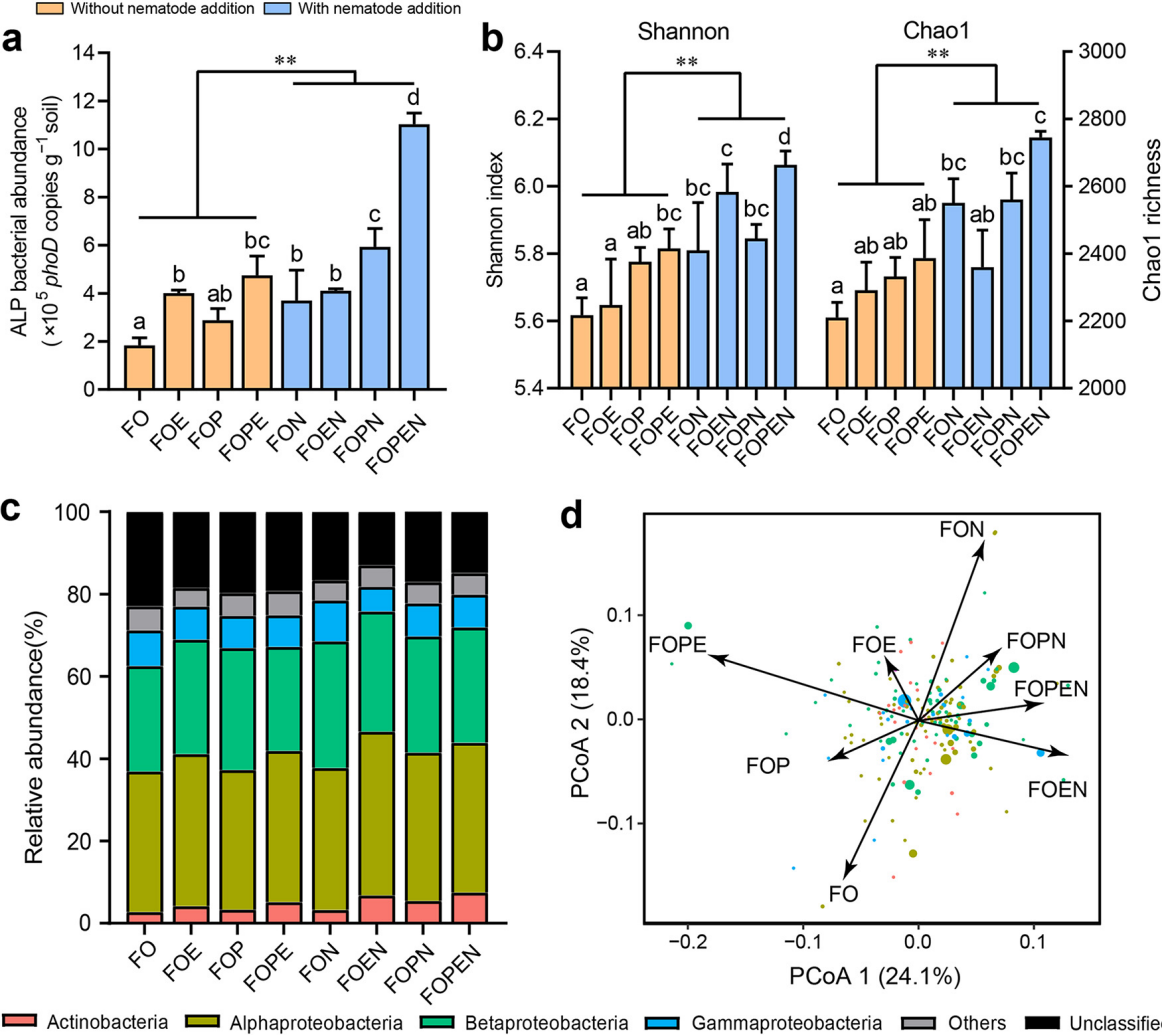
(a and b) Fertilization treatments alter the alkaline phosphomonoesterase (ALP)-producing bacterial abundance (a) and diversity (b) in the rhizosphere. Calculation of diversity and richness is based on the OTU matrix rarified to the same sequencing depth. Bars with different letters at the top are significantly different (*P *< 0.05) by Tukey’s honestly significantly difference (HSD) test. The ALP-producing bacterial abundance and diversity showed significant differences between treatments with and without nematode addition (**, *P *< 0.01). (c) Taxonomic compositions of the alkaline phosphomonoesterase (ALP)-producing bacterial communities in the rhizosphere at the phylum/class level. (d) Principal-coordinate analysis (PCoA) shows the dominant OTU (relative abundance > 0.1%) scores in the ALP-producing bacterial community based on the Bray-Curtis distances. Circle sizes indicate the abundances of ALP-producing bacterial taxa, and the colors are assigned to different phyla/classes. FO, chemical fertilizer with organic manure; FOE, FO plus earthworms; FOP, FO plus phosphate-solubilizing bacteria; FON, FO plus nematodes; FOPE, FO plus phosphate-solubilizing bacteria and earthworms; FOPN, FO plus phosphate-solubilizing bacteria and nematodes; FOEN, FO plus nematodes and earthworms; FOPEN, FO plus phosphate-solubilizing bacteria, earthworms, and nematodes.

The abundance, diversity, and composition of the rhizosphere ALP-producing bacterial community were quantified by quantitative PCR (qPCR) and profiled using Illumina sequencing of the *phoD* gene. We found that the copy numbers of ALP-producing bacteria were significantly higher under treatments with nematode addition (Fig. 1a) (*P *< 0.05), following a trend of FOPEN > FOPN > FOEN > FON. Similarly, the Shannon index and Chao1 richness of the ALP-producing bacterial community were significantly higher with nematode addition (Fig. 1b) (*P *< 0.05). The ALP-producing bacterial taxa were mostly affiliated with *Alphaproteobacteria* (36.1%), *Betaproteobacteria* (28.1%), *Gammaproteobacteria* (8.0%), and *Actinobacteria* (4.8%) (Fig. 1c). The canonical analysis of principal coordinates revealed that nematode addition exerted a significant impact on the structure of the ALP-producing bacterial community in the rhizosphere (*P *< 0.001) (Fig. 1d). Analysis of similarities (ANOSIM) confirmed the significance of these differences in the ALP-producing bacterial community between treatments with and without nematode addition (ANOSIM, *R *= 0.249, *P *= 0.015). The abundance (*r *= 0.759, *P *< 0.001), diversity (Shannon index: *r *= 0.863, *P *< 0.001; and Chao1 richness: *r *= 0.654, *P *= 0.001), and composition (*r *= 0.588, *P *= 0.003) of the ALP-producing bacterial community showed significant relationships with ALP activity. A Venn diagram indicated that a substantial fraction of bacterial OTUs (1,159) was commonly shared in the ALP-producing bacterial community present under treatments with and without nematode addition, representing 87.2% of the total sequences (Fig. S2a). These shared OTUs were dominated by the genera *Aquabacterium* (*Betaproteobacteria*, 20.4%, 68 OTUs), *Bradyrhizobium* (*Alphaproteobacteria*, 15.4%, 173 OTUs), *Pseudolabrys* (*Alphaproteobacteria*, 9.2%, 58 OTUs), *Mitsuaria* (*Betaproteobacteria*, 3.7%, 32 OTUs), and Pseudomonas (*Gammaproteobacteria*, 3.2%, 42 OTUs) (Fig. S2b). The genera *Aquabacterium*, *Pseudolabrys*, *Mitsuaria*, and Pseudomonas were enriched with nematode addition, while *Mesorhizobium* displayed an opposite pattern (Fig. S2c).

10.1128/mbio.03293-21.2 FIG S2 (a) Venn diagram showing the number of shared and unique ALP-producing bacterial OTUs in the rhizosphere without (orange) and with (blue) nematode addition. (b) Pie chart showing the taxonomic compositions of the shared OTUs at the genus level. The numbers in parentheses indicate the OTUs observed in each genus. (c) Heat map revealing OTUs enriched without and with nematode addition. The OTUs significantly enriched and depleted with nematode addition are represented by filled triangles (*P *< 0.05). Download FIG S2, TIF file, 1.3 MB. Copyright © 2022 Zheng et al. 2022 Zheng et al. https://creativecommons.org/licenses/by/4.0/ This content is distributed under the terms of the Creative Commons Attribution 4.0 International license.

### Nematode abundance and predation pressure.

**FIG 2:**
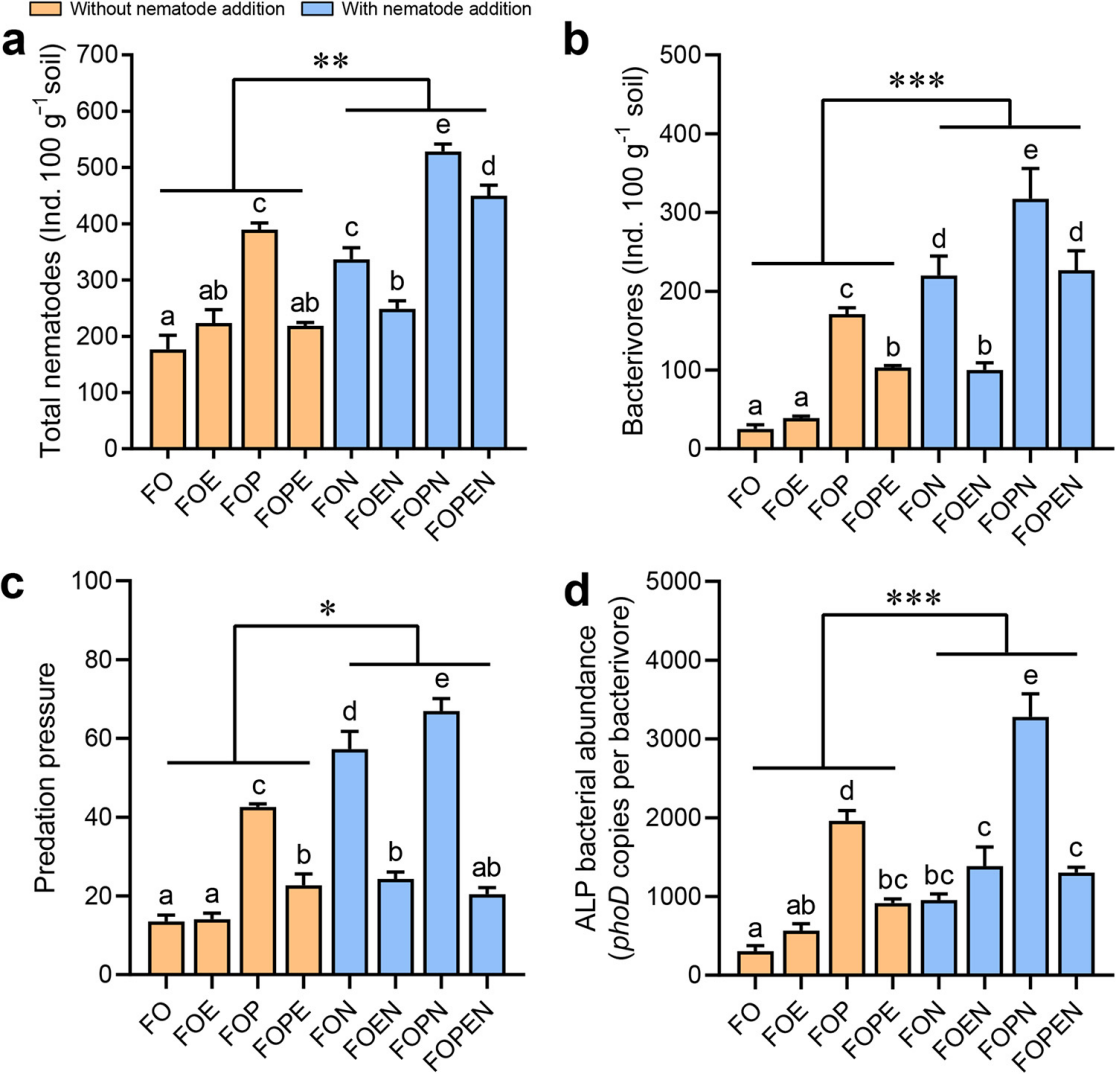
(a and b) The density of total nematodes (a) and bacterivorous nematodes (b) in the rhizosphere. (c) The inferred predation pressure of nematodes on the ALP-producing bacterial community. Predation pressure of bacterivorous nematodes on ALP-producing bacteria was calculated as the ratio of the number of bacterivorous nematodes to ALP-producing bacterial abundance. (d) The abundance of ALP-producing bacteria inside the body of bacterivorous nematodes. Bars with different letters at the top are significantly different (*P *< 0.05) by Tukey’s HSD test. ACP and ALP activities showed significant differences between treatments with (blue) and without nematode (orange) addition. FO, chemical fertilizer with organic manure; FOE, FO plus earthworms; FOP, FO plus phosphate-solubilizing bacteria; FON, FO plus nematodes; FOPE, FO plus phosphate-solubilizing bacteria and earthworms; FOPN, FO plus phosphate-solubilizing bacteria and nematodes; FOEN, FO plus nematodes and earthworms; FOPEN, FO plus phosphate-solubilizing bacteria, earthworms, and nematodes. ***, *P *< 0.001; **, *P *< 0.01; *, *P *< 0.05.

The total number of nematodes in the rhizosphere significantly varied across the fertilization treatments (*P *< 0.01). Overall, bacterivorous nematodes were the dominant group, accounting for 41.2% of all identified nematodes. The density of total nematodes and bacterivores increased by 55.1% and 92.2%, respectively, under treatments with nematode addition (Fig. 2a and b). As expected, the inferred predation pressure of bacterivorous nematodes on the ALP-producing bacterial community was significantly higher with nematode addition than without nematode addition (Fig. 2c) (*P *< 0.05). Similarly, the abundance of the *phoD* gene inside bacterivorous nematodes was significantly higher under treatments with nematode addition (1,370 ± 287 copies per bacterivore) than under treatments without it (937 ± 194 copies per bacterivore) (Fig. 2d) (*P *< 0.05). The density of total nematodes and bacterivores was significantly associated with the abundance (*r *= 0.654, *P *= 0.001 and *r *= 0.565, *P *= 0.004, respectively) and diversity of ALP-producing bacterial populations (Shannon index: *r *= 0.535, *P *= 0.007 and *r *= 0.512, *P *= 0.011; Chao1 richness: *r *= 0.514, *P *= 0.010 and *r *= 0.493, *P *= 0.014), as well as ALP activity (*r *= 0.525, *P *= 0.008 and *r *= 0.604, *P *= 0.002).

### Effect of nematode addition on ALP-producing bacterial networks.

**FIG 3:**
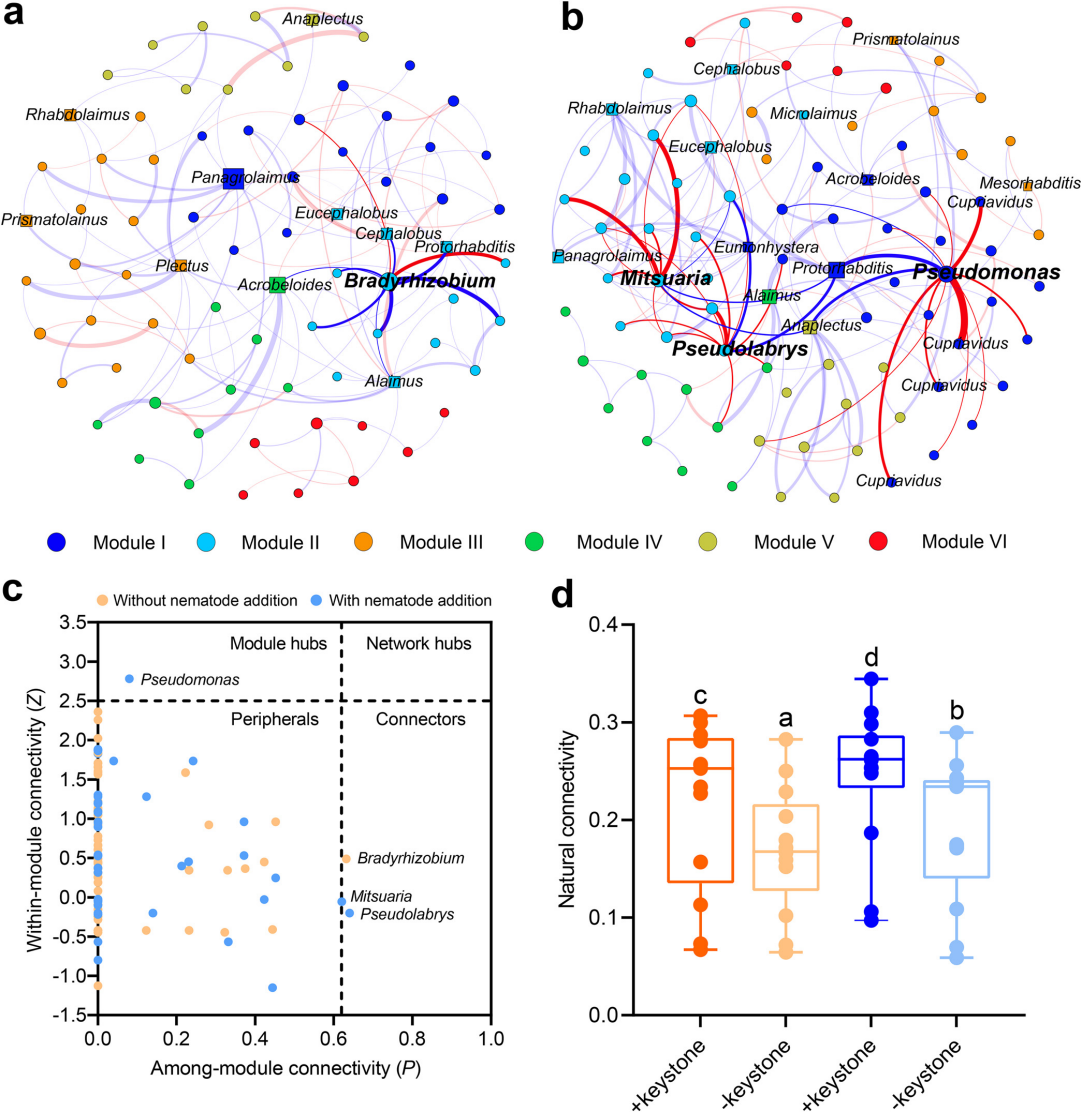
(a and b) The cooccurrence networks of the alkaline phosphomonoesterase (ALP)-producing bacterial community (circles) and bacterivorous nematodes (squares) in the rhizosphere between treatments without (a) and with (b) nematode addition are colored based on modules. A connection stands for a strong (Spearman’s *r *> 0.7 or < −0.7) and significant (*P *< 0.01) correlation. Modules I to VI in the ALP-producing bacterial networks represent the six clusters of closely interconnected nodes. For each panel, the names of bacterial keystone taxa (module hubs and connectors) and their associated edges in the networks are in bold, while the names of connected nodes (*Cupriavidus*) of keystone taxon Pseudomonas are in regular type. Based on network analysis, the Pseudomonas and *Cupriavidus* taxa are further selected in the competition experiments. The size of each node is proportional to the number of connections (degree), and the thickness of each connection between two nodes (edge) is proportional to the value of Spearman’s correlation coefficients. The blue edges indicate positive correlations between two individual nodes, and the red edges indicate negative correlations. (c) A *Z-P* plot shows the distribution of nodes based on their topological roles in the ALP-producing bacterial networks between treatments with and without nematode addition. Each symbol represents an OTU in the networks, and the keystone taxa (module hubs and connectors) are labeled. (d) The network robustness between treatments with and without nematode addition is indicated by the natural connectivity. The natural connectivity is also calculated to assess the influence of keystone taxa on the network robustness. Bars with different letters at the top are significantly different by Tukey’s HSD test (*P *< 0.05).

Cooccurrence networks differed between treatments with and without nematode addition, including the ratios of positive and negative correlations, average connectivity, and modularity values (Fig. 3; Table S2). Although the number of positive correlations was much higher than that of negative correlations in both networks, the ratio of negative to positive correlation increased with nematode addition (48.6%), compared to without nematode addition (25.9%). This is also reflected in greater average connectivity and graph density (from 3.573 and 0.049 to 3.402 and 0.044, respectively).

10.1128/mbio.03293-21.7 TABLE S2 Topological properties of ALP-producing bacterial cooccurrence networks with (+N) and without (−N) nematode addition. Download Table S2, DOCX file, 0.02 MB. Copyright © 2022 Zheng et al. 2022 Zheng et al. https://creativecommons.org/licenses/by/4.0/ This content is distributed under the terms of the Creative Commons Attribution 4.0 International license.

Based on the topological analysis of the nodes, nematode addition was related to an increase in the number of potential keystone taxa (connectors and module hubs) in the ALP-producing bacterial community. For the network without nematode addition, the genus *Bradyrhizobium* (0.29%) was detected as a connector and positively associated with other members in the module (Fig. 3a and c; Table 1). Module II was negatively associated with TN, AN, ALP-producing bacterial abundance, and ALP activity (Fig. S3a) (*P *< 0.05). For the network with nematode addition, the genus Pseudomonas (0.20%) was identified as the module hub, while the genera *Pseudolabrys* (0.12%) and *Mitsuaria* (0.11%) were designated as connectors (Fig. 3b and c; Table 1). These potential keystone taxa were negatively associated with linked nodes in the individual network modules, but their relative abundances displayed significantly positive relationships with the density of bacterivorous nematodes and ALP activity (Table 1) (*P* < 0.05). Modules I and II were positively correlated with TN and AN, as well as ALP-producing bacterial diversity, abundance, the total number of nematodes, and ALP activity (Fig. S3b) (*P *< 0.05). The natural connectivity was significantly higher with nematode addition than without nematode addition (Fig. 3d) (*P *< 0.05). The *in silico* removal of these potential keystone taxa in both networks resulted in a significant decrease (*P *< 0.05) of the natural network connectivity.

**TABLE 1:** Keystone taxa in the ALP-producing bacterial networks with and without nematode addition

Network^*a*^	OTUID	Role	Module	Degree	Abundance (%)	Class	Genus	*Z* ^*b*^	*P* ^*b*^	Nematode density^*c*^	ALP activity
−N	OTU33	Connector	II	10	0.29	*Alphaproteobacteria*	*Bradyrhizobium*	0.48	0.63	0.275	0.377
+N	OTU103	Module hub	I	13	0.20	*Gammaproteobacteria*	Pseudomonas	2.78	0.08	0.643**	0.81**
	OTU178	Connector	II	11	0.11	*Betaproteobacteria*	*Mitsuaria*	−0.05	0.62	0.58**	0.64**
	OTU210	Connector	II	11	0.12	*Alphaproteobacteria*	*Pseudolabrys*	−0.20	0.64	0.56**	0.76**

a −N, without nematode addition; +N, with nematode addition.

b The topological role of each node is determined according to the within-module connectivity *Z* value and the among-module connectivity *P* value.

c Nematode density, density of bacterivorous nematodes.

10.1128/mbio.03293-21.3 FIG S3 Correlation matrix based on Pearson’s rank correlation coefficients between soil characteristics, nematodes, ALP-producing bacterial community, and soil enzymatic activities without (a) and with (b) nematode addition. Soil properties include pH, soil organic carbon (SOC), total nitrogen (TN), total phosphorus (TP), total potassium (TK), alkali-hydrolyzable nitrogen (AN), available phosphorus (AP), and available potassium (AK). The ALP-producing bacterial community includes the abundance, diversity, composition, and cooccurrence network. The abundance is characterized by the *phoD* gene copy number, the diversity is characterized by the Shannon index and Chao1 richness, and the composition is characterized by the first principal coordinates (PCoA1 explained 24.1% of the variations). The modules with keystone taxa in the networks without and with nematode addition are shown. The numbers in parentheses indicate the nodes observed in each module. The size and intensity of color for each circle represent the strength of the correlation, such that the larger, darker circles demonstrate a strong correlation. Blue colors indicate positive correlation coefficients, and red colors indicate negative correlations. ACP, acid phosphomonoesterase; ALP, alkaline phosphomonoesterase. Download FIG S3, TIF file, 2.9 MB. Copyright © 2022 Zheng et al. 2022 Zheng et al. https://creativecommons.org/licenses/by/4.0/ This content is distributed under the terms of the Creative Commons Attribution 4.0 International license.

### Integrating soil physicochemical properties, nematodes, and the ALP-producing bacterial community with ALP activity.

**FIG 4:**
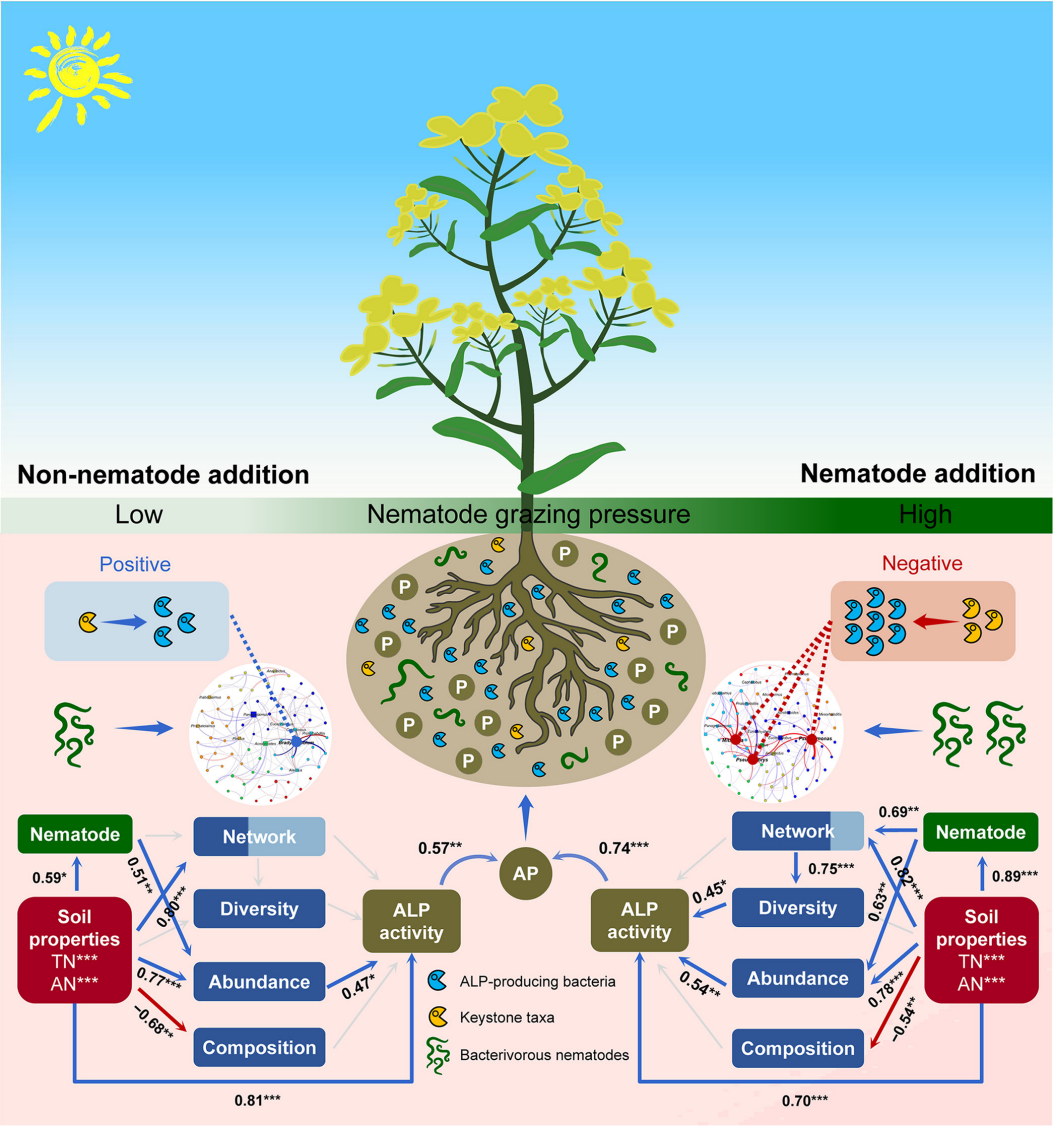
Structural equation modeling overview of the mechanism of soil properties and alkaline phosphomonoesterase (ALP)-producing bacterial community in mediating ALP activity between treatments with and without nematode addition. Soil properties include total nitrogen (TN) and available nitrogen (AN). The nematode assemblage is represented by the number of bacterivorous nematodes. The ALP-producing bacterial community includes the abundance (copy number of *phoD* gene), diversity (Shannon index), composition (first principal coordinates, PCoA1), and cooccurrence network. The network is represented by the module eigengenes that are significantly related to ALP activity. The contributions of the module with or without keystone taxa are indicated by dark or light shading, respectively. Blue lines indicate positive relationships, while red lines indicate negative relationships. The width of arrows indicates the strength of the significant standardized path coefficients, while paths with nonsignificant coefficients are represented by gray lines. ***, *P *< 0.001; **, *P *< 0.01; *, *P *< 0.05.

Random forest modeling revealed that AN (19.9%, *P < *0.01) and TN (17.4%, *P < *0.01) significantly predicted ALP activity. Furthermore, the diversity (14.6%, *P *< 0.01), abundance (8.4%, *P < *0.05), composition (6.9%, *P *< 0.05), and network (6.8%, *P < *0.05) of the ALP-producing bacterial community, as well as bacterivorous nematodes (12.0%, *P *< 0.01), significantly influenced ALP activity (Fig. S4). Structural equation modeling further suggested that soil properties and ALP-producing bacterial abundance had stronger effects on ALP activity without nematode addition than with nematode addition (*P < *0.001). However, the network with ALP-producing bacterial taxa was positively associated with the ALP-producing bacterial diversity and indirectly linked with ALP activity when nematodes were added (Fig. 4) (*P < *0.001).

10.1128/mbio.03293-21.4 FIG S4 Mean predictor importance (% of increased mean square error [MSE]) of soil properties, alkaline phosphomonoesterase (ALP)-producing bacterial community, and bacterivorous nematodes for ALP activity based on random forest modeling. Soil properties include pH, soil organic carbon (SOC), total nitrogen (TN), total phosphorus (TP), total potassium (TK), available nitrogen (AN), availability phosphorus (AP), and available potassium (AK). The ALP-producing bacterial community includes the abundance, diversity, composition, and cooccurrence network. The abundance is characterized by the *phoD* gene copy number, the diversity by the Shannon index, and the composition by the first principal coordinates (PCoA1 explained 24.1% of the variations). The network is represented by the module eigengenes that are significantly related to ALP activity. The model for ALP activity was significant at the 0.01 level (*R*^2^ = 0.68). The significance level of each predictor is as follows: *, *P *< 0.05; **, *P *< 0.01. Download FIG S4, TIF file, 0.3 MB. Copyright © 2022 Zheng et al. 2022 Zheng et al. https://creativecommons.org/licenses/by/4.0/ This content is distributed under the terms of the Creative Commons Attribution 4.0 International license.

### Bacterial competition assay in culture media.

**FIG 5:**
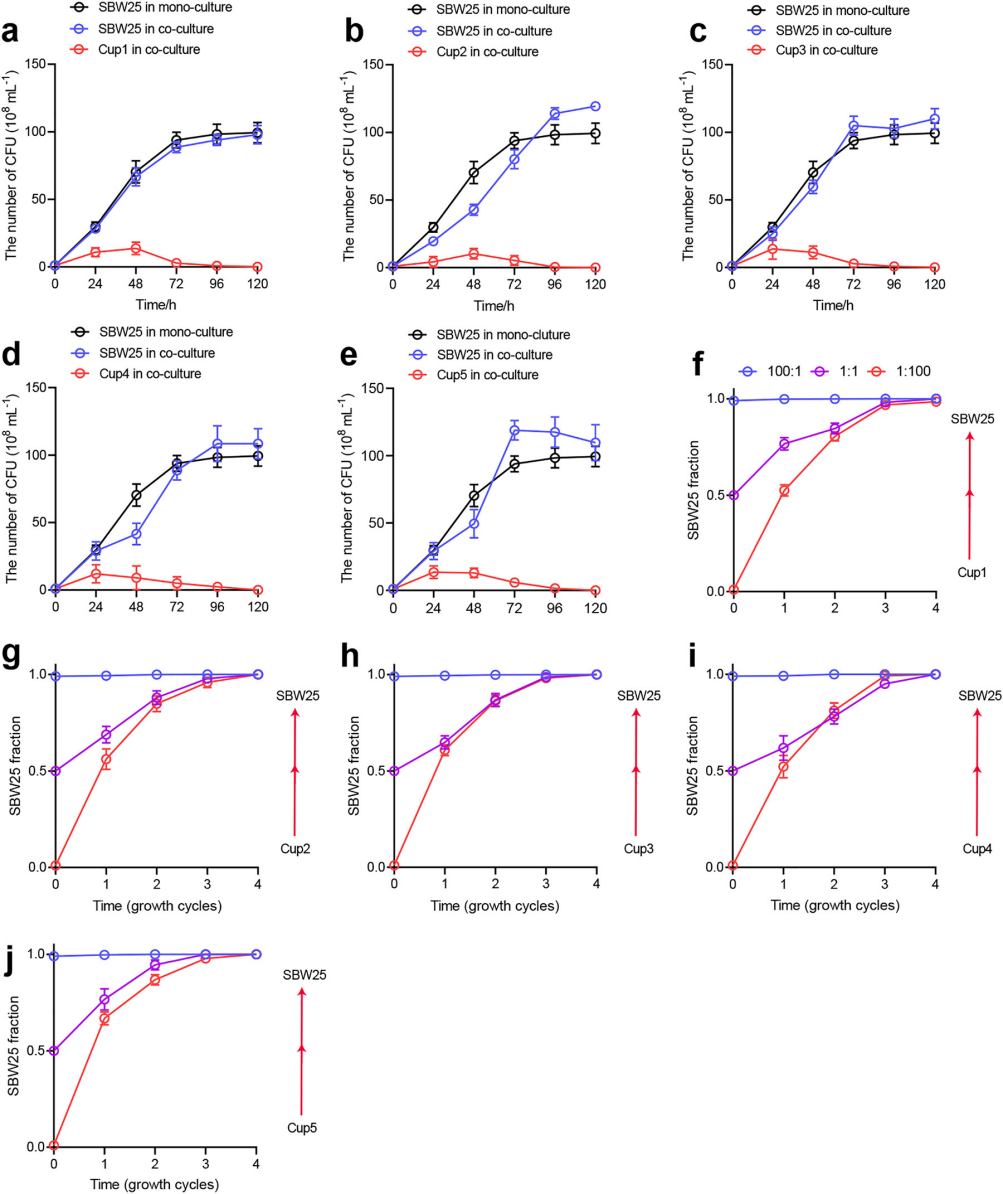
Defining the bacterial competitive interactions in liquid microcosms. (a to e) Compositional changes of P. fluorescens SBW25 and five *Cupriavidus* strains in pairwise cocultures over 5 days with shaking condition. (f to J) Changes in relative abundance of P. fluorescens SBW25 and five *Cupriavidus* strains at three different initial fractions of 1:1 (pink), 1:100 (red), and 100:1 (blue). The *y* axis indicates the fraction of one of the competing species. Red arrows on the right of each panel indicate the qualitative competitive outcome. Error bars represent the standard deviation of the fractions, based on colony counts averaged across 6 replicates.

To biologically validate the observed negative correlations between potential keystone taxa (Pseudomonas) and other connected members (*Cupriavidus*) in the network with nematode addition, we performed a series of bacterial competition experiments. We used the model bacterium Pseudomonas fluorescens SBW25 and a set of five soil-dwelling *Cupriavidus* sp. strains in agar plate and liquid cultures. The agar plate assays showed that SBW25 exhibited clear inhibition zones against Cupriavidus necator (Cup1, Cup2, Cup3) and Cupriavidus taiwanensis (Cup4, Cup5) (Fig. S5a). In liquid culture, the monocultures revealed that SBW25 was able to grow at higher cell densities and rates than the five *Cupriavidus* strains (Fig. S5b and c). In particular, SBW25 was antagonistic to *C. taiwanensis* and C. necator, with the mean interaction indices lower than 1 (Fig. S5d), and the five *Cupriavidus* strains tested went extinct at some point throughout the experiment (Fig. 5a to e). In line with these results, we observed that SBW25 was able to competitively exclude both *C. taiwanensis* and C. necator at three different initial ratios of 1:1, 1:100, and 100:1 but that *Cupriavidus* sp. strains were unable to invade SBW25 in the competitive experiment (Fig. 5f to j). After cocultures in static microcosms, we found that SBW25 diversity (Shannon index) was significantly (*P *< 0.05) higher than that in monocultures (Fig. S5e). Taken together, our results validate *in vitro* the ability of SBW25 to negatively affect the growth of *C. taiwanensis* and C. necator.

10.1128/mbio.03293-21.5 FIG S5 Inhibitory effects of Pseudomonas fluorescens SBW25 on the growth of Cupriavidus necator (Cup1, Cup2, and Cup3) and Cupriavidus taiwanensis (Cup4 and Cup5). (a) SBW25 exhibits clear inhibition zones against Cupriavidus necator and Cupriavidus taiwanensis. (b and c) Cell density (b) and growth rates (c) of Pseudomonas fluorescens SBW25 and a set of five soil-dwelling *Cupriavidus* strains in monocultures. (d) Diversity of P. fluorescens SBW25 in the presence of *Cupriavidus* sp. in the heterogeneous environment (static microcosms in 6 mL KB medium with 5% sterile soil suspension at 28°C after 4 days). SBW25 diversity is indicated by the number of unique colony morphotypes. (e) Mean interaction indices, calculated as the ratio of observed to expected cell densities, between Pseudomonas fluorescens SBW25 and Cupriavidus necator or Cupriavidus taiwanensis. A ratio value of 1 indicates additive growth, that is, no interaction; values of <1 indicate potential antagonistic interactions; and values of >1 indicate potential facilitation. Lowercase indicates significant difference between groups (*P *< 0.05) via Tukey’s *post hoc* tests, based on colony counts averaged across 6 replicates. Box plots indicate median (middle line), 25th, and 75th percentile (box) and 5th and 95th percentile (whiskers). Download FIG S5, TIF file, 2 MB. Copyright © 2022 Zheng et al. 2022 Zheng et al. https://creativecommons.org/licenses/by/4.0/ This content is distributed under the terms of the Creative Commons Attribution 4.0 International license.

**FIG 6:**
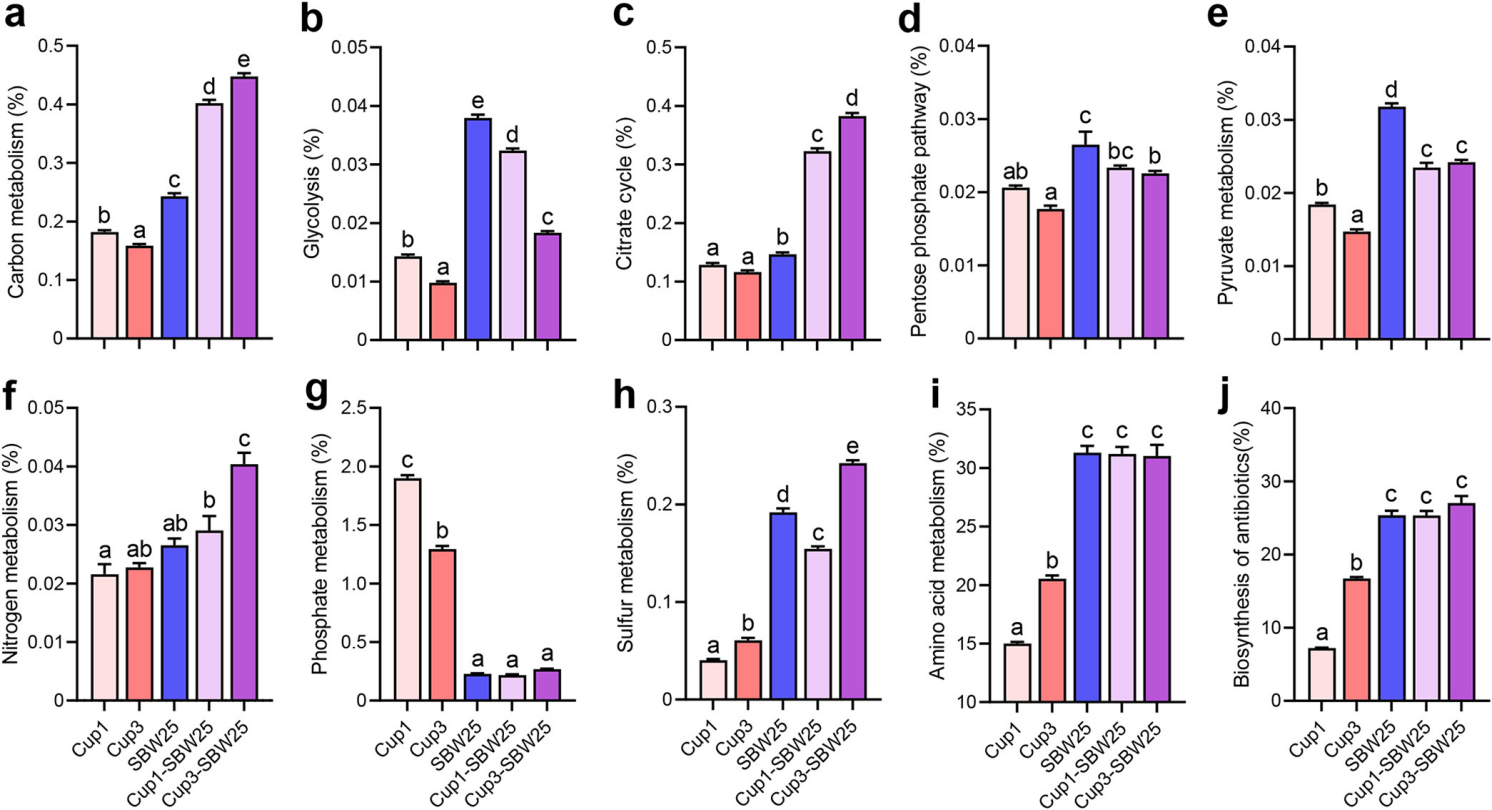
Metabolic intensity involved in the carbon (a to e), nitrogen (f), sulfur (g), phosphate (h), and amino acid (i) metabolisms and biosynthesis of antibiotics (j) based on an untargeted metabolomics approach. The carbon metabolism contains four pathway modules, such as citrate cycle, glycolysis/gluconeogenesis, pentose phosphate pathway, and pyruvate metabolism. Bars with different letters at the top indicate significant differences as revealed by Tukey’s HSD tests (*P *< 0.05). SBW25, P. fluorescens SBW25 in monoculture; Cup1, Cupriavidus necator in monoculture; Cup3, Cupriavidus taiwanensis in monoculture; Cup1-SBW25, Cupriavidus necator and P. fluorescens SBW25 in coculture; Cup3-SBW25, Cupriavidus taiwanensis and P. fluorescens SBW25 in coculture.

We further determined the potential suppressive impact of SBW25 on *C. taiwanensis* and C. necator by using an untargeted metabolomics approach. Analysis of the metabolic profiles revealed that carbon, nitrogen, sulfur, and amino acid metabolisms of SBW25 were significantly higher than those of *C. taiwanensis* and C. necator in monoculture (Fig. 6a to i) (*P *< 0.05). The accumulation of antibiotic biosynthesis in SBW25 was also significantly higher (Fig. 6j) (*P *< 0.05). The relative abundances of dominant pathway modules related to carbon metabolism (*P *< 0.001) followed an increasing trend, including citrate cycle, glycolysis/gluconeogenesis, pyruvate metabolism, and pentose phosphate pathway (*P *< 0.05). In contrast, the phosphate metabolism was significantly lower in SBW25 than in *C. taiwanensis* and C. necator (Fig. 6g).

## DISCUSSION

### Nematode addition significantly alters the ALP-producing bacterial community.

Our study showed that chemical and organic fertilizers combined with nematode addition significantly improved the overall nutrient availability (TN and AN) in the plant rhizosphere. The increase in N availability likely suggests that nematode predation “releases” N due to differences in C/N ratios within microbes and nematode cells (20). Further, we clearly revealed that nematode feeding on specific microbial taxa led to changes in the overall community structure and higher ALP-producing bacterial abundance and diversity in the rhizosphere. A quantitative review argues that bacterivorous nematodes can reduce microbial biomass by 16% and bacterial abundance by 17% with direct implications for nutrient cycling and community dynamics (21). The impact of nematode predation is not random, as bacterium-feeding nematodes have preferential feeding responses to specific food sources based on their divergent buccal morphology (22, 23). Hence, small-sized bacteria are presumably easier to swallow through the narrow buccal cavity, allowing more efficient ingestion and nutrient acquisition. Alternatively, nematodes have a chemosensory system as vertebrates and insects, which enables them to choose preferred food to maintain metabolic functions (24). Selective feeding traits of nematodes may facilitate the population turnover that maintains the ALP-producing bacterial community and also may affect rates of species diversification through the evolution of novel predator resistance/avoidance strategies (23, 24). Our previous study provides other pieces of evidence that selective predation benefits nematode fitness and exhibits these positive impacts on the dynamic alterations in the ALP-producing bacterial community (11). It is worth noting that the presence of earthworms significantly reduced the number of bacterivorous nematodes and the predation pressure of bacterivores on the ALP-producing bacterial community under FOEN treatment compared to FON treatment. Earthworms have been widely reported to negatively influence the density of nematodes owing to direct predation on soil nematodes and indirect modifications in soil properties related to earthworm activity (25, 26).

### Nematode predation favors ALP-producing bacterial diversity via subsequent competitive interactions.

Major differences in network structure were observed in the presence or absence of nematode addition. Our findings from the field experiment combined with pairwise coculture assays suggested that higher pressure of nematode predation can induce competitive interactions between keystone species and other members within the community. The combined influence of these ecological interactions likely promoted the diversity and stability of the rhizosphere ALP-producing bacterial community. The higher population density of bacterivorous nematodes and the predation pressure linked with distinct keystone taxa and enhanced the network robustness of the ALP-producing bacterial community, as evidenced by greater network connectivity. Theoretically, microbiome diversity and stability are expected to be greater in systems with higher interspecific competition (19, 27). We identified Pseudomonas, *Mitsuaria*, and *Pseudolabrys* as potential keystone taxa exhibiting competitive interaction with other taxa in their respective modules. Worth mentioning, the inhibitory effect of microbial competition is likely driven not only by the production of antibiotics and secondary metabolites (28) but also by competition for resources and space (29). In the case of keystone taxa, these often form a central clustering pattern with other members within the community, largely contributing to network robustness (30, 31). As such, keystone taxa are expected to exert crucial roles in maintaining the organizational integrity and stability of the microbiome community (11, 13). By simulating the removal of these keystone taxa in our data, we found the network robustness of the rhizosphere ALP-producing bacteria to dramatically decrease irrespective of nematode addition. Similar results have also been reported elsewhere, illustrating the influences of keystone species for network stability and their removal cascade effects on microbiome diversity and composition (32, 33). However, caution is warranted when inferring the stimulatory effect of predation-induced competitive interactions on the ALP-producing bacterial community. We found that the relative abundance of Pseudomonas was positively correlated with the relative abundance of *Anaplectus* and *Protorhabditis* in the network (Fig. 3b). In this case, this particular predator-prey relationship indicated that the two nematodes feeding on the keystone taxa may contribute to the lower abundance of ALP-producing bacteria. Therefore, further analytical developments and empirical evidence are required to confirm our findings and finely dissect this relationship.

### Nematode addition results in positive associations between ALP-producing bacterial diversity and ALP activity.

The soil bacterial community plays major roles in diverse ecosystem functions, particularly in modulating the biogeochemical cycling of essential nutrients (5, 34). These functions, however, are often studied in the absence of consideration of trophic cascades and ecological interactions in the soil system. Here, we showed that the abundance and diversity of ALP-producing bacterial populations were significantly correlated with ALP activity under treatments that received nematode addition. The ALP-producing bacteria can efficiently act on P mineralization once in a local environment that provides optimum nutrients. This “stimulatory effect” may result from a high grazing pressure driven by bacterivorous nematodes feeding on specific keystone taxa (11). However, establishing a causal relationship between microbiome composition and functioning remains challenging. A recent meta-analysis has reported that microbial richness is positively correlated with ecosystem functioning, such as P mineralization (35). Keystone taxa often have profound contributions to the relationships between microbiome diversity and ecosystem functioning by regulating species interactions in communities (14, 36). We therefore postulated that nematode addition drove keystone taxa to compete with the connected members via high predation pressure of bacterivores and thereby facilitated the significantly positive associations between bacterial diversity and P mineralization. Keystone taxa have been suggested to engage in antagonistic relationships and alter the abundance of their partners, and this can directly translate into a significant impact on community structure and functional performance (13). The strength of the impact of keystone taxa in microbial diversity and composition is pertinent to the rate of community functional change (37, 38). Collectively, our findings provide new insights into how nematode predation drives ecological functionality and the relevance of keystone taxa for the biodiversity-functioning relationships.

### Conclusions.

This study provides empirical evidence to support the important role of nematode predation in shaping the rhizosphere microbiome community. This modulation subsequently leads to functional changes affecting P availability. In particular, predation pressure was shown to alter competitive interactions between keystone taxa and members within the community, resulting in changes in the ALP-producing bacterial community and ALP activity. Predation was positively related to the ALP-producing bacterial abundance and significantly structured the overall rhizosphere community composition. Taken together, our results provide new insights into microbially mediated mechanisms of competitive interaction induced by nematode predation in enhancing P availability in the plant rhizosphere. We advocate for the need to consider biotic interactions and trophic cascades in future studies aiming at elucidating the rates and fates of nutrient cycling dynamics in soils.

## MATERIALS AND METHODS

### Site description and experimental design.

The long-term field experiment was established at the Red Soil Ecological Experimental Station of the Chinese Academy of Sciences (28°15′N, 116°55′E), Yingtan County, Jiangxi Province. The climate is defined as warm and humid monsoon, with an average annual temperature and precipitation of 18.1°C and 1,785 mm, respectively. The soil in the area is developed from Quaternary red clay and is classified as Ferric Acrisol according to the FAO classification system. The experiment design included eight treatments with three replicated plots (5 m by 4 m), as follows: (i) FO, chemical fertilizer with organic manure; (ii) FOE, FO plus earthworms; (iii) FOP, FO plus phosphate-solubilizing bacterium; (iv) FON, FO plus nematodes; (v) FOPE, FO plus phosphate-solubilizing bacterium and earthworms; (vi) FOPN, FO plus phosphate-solubilizing bacterium and nematodes; (vii) FOEN, FO plus nematodes and earthworms; and (viii) FOPEN, FO plus phosphate-solubilizing bacterium, earthworms, and nematodes. These eight treatments were divided into two groups: one with (FON, FOEN, FOPN, FOPEN) and one without (FO, FOE, FOP, FOPE) nematode addition. Since 2011, the chemical fertilizers, organic manure, phosphate-solubilizing bacterium, nematode, and earthworm had been applied annually under the rotation system of summer sweet potato (Ipomoea batatas L.) and winter rapeseed (Brassica napus L.). The chemical fertilizers were applied using 152.75 kg N ha^−1^ year^−1^, 116.20 kg P_2_O_5_ ha^−1^ year^−1^, and 166.32 kg K_2_O ha^−1^ year^−1^. Organic manure was applied using composted pig manure (11,250 kg ha^−1^ year^−1^), containing an average total nitrogen of 13.6 g kg^−1^, total phosphorus of 2.67 g kg^−1^, and total potassium of 1.51 g kg^−1^ based on dry matter mass. The inoculum of phosphate-solubilizing bacterium was based on the *Mesorhizobium* sp. isolated from red clay soils (Ultisols) prepared in an aqueous solution and inoculated at a density of 5 × 10^12^ CFU m^−2^ year^−1^ (39). Earthworms (Eisenia fetida) were obtained from a farm in Yingtan and added at a density of ca. 1,000 individuals m^−2^ year^−1^ (40). Nematode addition was based on a mixed population mostly (80.8%) composed of bacterium-feeding species (the dominant genus *Protorhabditis*, 92.1% in all bacterivores) collected at the local soil site (41). Briefly, 3 kg of the soil in the plot was mixed with 30 kg of dried pig manure and incubated at 20°C for 28 days. The mixed soils were added back to the plot containing a nematode density of ca. 50 individuals g^−1^ year^−1^. The reduced amount of N in composted pig manure was supplemented to equal that of the FO treatment. The chemical fertilizers and organic manure were annually applied as basal fertilization before the cultivation with sweet potato, and additional chemical fertilizers were equally applied in all plots before the cultivation with rapeseed.

### Rhizosphere sampling and physicochemical properties.

Rhizosphere samples were collected in the rapeseed phase of the rotation system after harvest in April 2018. Rhizosphere soils were taken by sampling rapeseed plants in each plot, kept on ice, and immediately (<24 h) transported to the laboratory. After gently shaking the roots, the adhering rhizosphere soils were collected with a brush and then passed through a 4-mm sieve to remove plant materials and other debris. Rhizosphere samples were subdivided into three subsamples and subjected to analysis for soil physicochemical properties, nematode assemblage, and microbiome profiling.

Soil pH was determined using a glass electrode in a 1:2.5 (wt/vol) soil-to-water solution. Soil organic carbon (SOC) was determined using the potassium dichromate method (42). The total nitrogen (TN) was measured by the Kjeldahl method (43), and alkali-hydrolyzable nitrogen (AN) was measured by the alkaline hydrolysis diffusion method (44). Total phosphorus (TP) was digested with HF-HClO_4_, and available phosphorus (AP) was extracted with sodium carbonate and sodium bicarbonate and further determined with the molybdenum blue method (45, 46). Total potassium (TK) was digested with HF-HClO_4_, and available potassium (AK) was extracted with ammonium acetate, which were detected by atomic absorption spectrophotometer (47). The acid and alkaline phosphomonoesterase (ACP and ALP) activities were determined using *p*-nitrophenyl (p-NP) phosphate as the substrate with the buffer adjusted to pH 6.5 and 11.0, respectively (48). After incubation, the absorption was measured at 405 nm. Both ACP and ALP activities were expressed as mg p-NP g^−1^ soil h^−1^.

### Quantitative PCR and Illumina sequencing of the bacterial *phoD* gene.

To address question *i* from the introduction, we determined the abundance, diversity, and composition of the ALP-producing bacterial community by quantitative PCR and Illumina sequencing of the *phoD* gene. Total DNA was extracted from 0.5 g of fresh rhizosphere soil using the Ultraclean Soil DNA isolation kit (MoBio, CA, USA), according to the manufacturer’s protocol. The quality and quantity of total DNA in each sample were determined using a NanoDrop ND-2000 spectrophotometer. qPCR of the bacterial *phoD* gene was performed using the primers ALPS-F730 and ALPS-R1101 (49). Each sample was amplified in a 20-μL reaction mixture containing 0.5 μL of each primer, 10 μL of 2× SYBR Premix Ex Taq, 1 μL of template DNA, and 8 μL of double-distilled water (ddH_2_O). Thermocycling conditions were as follows: 3 min of initial denaturation at 95°C, followed by 40 cycles of 95°C for 30 s, 60°C for 5 s, and at 72°C for 34 s, with a final extension of 72°C for 10 min. The qPCR was performed in triplicate, and amplification efficiencies (E) of >97% were obtained with *r*^2^ values of >0.99. The abundance of ALP-producing bacteria was expressed as the *phoD* gene copy number per gram of dry soil.

High-throughput sequencing was performed on an Illumina MiSeq platform for the amplicon fragment of the *phoD* gene obtained by using the same primer pair used for the qPCR. Raw sequences were quality screened and trimmed using QIIME (version 1.9.1) (50). Sequences that fully matched the barcodes were kept, demultiplexed, and quality trimmed for further analysis. Chimeric sequences were removed using the UCHIME algorithm within the USEARCH package (51). The remaining sequences were further screened for frameshifts using HMM-FRAME (52). Quality sequences were compared against the NCBI nonredundant nucleotide database using BLASTN. Sequences were clustered to provide similarity-based operational taxonomic units (OTUs) using CD-HIT-EST at 97% of nucleotide identity (53). Alpha diversity (Shannon index and Chao1 richness) and community relatedness of the ALP-producing bacterial community were calculated after rarefaction of all samples to the same sequencing depth of 29,707 sequences.

### Nematode assemblages.

To address question *i*, nematodes were extracted from soil samples using the shallow dish method (41). Briefly, 100 g of fresh soil was placed evenly on a double cotton filter over a stainless steel sieve tray (10 mesh). The sieve tray was put in a matching shallow dish, and distilled water was added to the dish to submerge the sample. The water in the shallow dish was filtered through two stacked sieves (500 mesh) after 2 days. All nematodes left on the surface of the sieves were collected into plastic petri dishes. Nematodes were counted using a dissecting microscope and expressed as the number of individuals 100 g^−1 ^dry soil. The bacterivores were identified based on known feeding habits, stoma, and esophageal morphology (54).

The bacterivorous nematodes were collected separately into sterile phosphate buffer (pH 7.0) under a dissecting microscope based on their morphological properties. These collected bacterivores were introduced into a 2% sodium hypochlorite solution for 30 s to prevent microbial interference on the body surface and then washed five times with sterile distilled water. To examine the predation of bacterivores on ALP-producing bacteria, all individuals of bacterivores in 100 g of fresh soil were collected and transferred into a 2-mL centrifuge tube for DNA extraction. The copy number of the ALP-producing bacterial *phoD* gene inside the body of bacterivorous nematodes were quantified via qPCR. No amplification was detected in the final wash water, indicating the absence of external contamination and efficiency of nematode surface sterilization.

### Cooccurrence network analysis and identification of potential keystone taxa.

To address question *ii*, we conducted network analysis to evaluate the influence of nematode addition on network structure and identify the potential keystone taxa. Cooccurrence networks of the ALP-producing bacterial community and bacterivorous nematodes were obtained by constructing a correlation matrix that calculated all possible pairwise Spearman’s rank correlations between pairs of taxa. The soil samples with (FON, FOEN, FOPN, and FOPEN) and without (FO, FOE, FOP, and FOPE) nematode addition were analyzed separately to evaluate the potential influence of nematode addition on network configurations. A valid cooccurrence was considered statistically robust based on a Spearman’s correlation coefficient (*r*) of >0.7 or < −0.7 and a *P *of <0.01. The *P* values were adjusted for multiple tests using the Benjamini-Hochberg method to prevent false-positive results (55). The topological features of average path length, graph density, network diameter, average clustering coefficient, average connectivity, and modularity were calculated individually for each network. The topological role of each node (OTUs) was determined based on the within-module connectivity *Z* score and the among-module connectivity *P* score (56). All nodes were sorted into four subcategories: peripherals (*Z *< 2.5 and *P *< 0.62), module hubs (*Z* > 2.5 and *P *< 0.62), connectors (*Z *< 2.5 and *P *> 0.62), and network hubs (*Z *> 2.5 and *P *> 0.62). The nodes with a high value of *Z* or *P* (module hubs, connectors, and network hubs) were classified as potential keystone taxa. The module eigengene was used to summarize the closely connected members within a module in the network (57). The singular value decomposition of the module expression data matrix was used to represent the module eigengene. The module eigengene was designated as the first principal component of each module (58). Network analyses were performed using the igraph (59), vegan (60), and Hmisc (61) packages in R. Networks were visualized using the software Gephi with the Fruchterman-Reingold layout (62).

### Bacterial competition in microcosms.

To address question *ii*, we validated experimentally the competitive correlations between keystone taxa (here, we selected Pseudomonas) and other connected members (here, we selected *Cupriavidus*) in the networks. We used five *Cupriavidus* sp. strains with a strong ability of dissolving phosphorus (63), including two *C. taiwanensis* strain (Cup1 and Cup2) and three C. necator strains (Cup3, Cup4, and Cup5). The five *Cupriavidus* sp. strains were isolated from rhizosphere soil in the field plots according to previous protocols (64). We also used the common model bacterium Pseudomonas fluorescens SBW25, which possesses the *phoD* gene (65). The SBW25 strain can rapidly diversify in static liquid microcosms (heterogeneous environmental conditions), generating three dominant colony morphologies (named wild-type smooth, wrinkly spreader, and fuzzy spreader) (66).

Fresh soil (10 g dry weight) was diluted 1:9 in 0.9% NaCl in sterilized water, vortexed at 200 rpm for 30 min, and left steady for 1 h to allow coarse particles to sediment. The supernatant was collected and filtered through a 0.22-μm filter using sterile 50-mL tubes. Individual and coculture treatments were performed using King’s B (KB) medium containing 5% sterile soil suspension. To estimate the population density, bacterial growth was calculated as CFU mL^−1^. Before the competition experiment, single colonies were grown separately in KB medium for 24 h. The experiment consisted of dual-species cultures inoculated at the same population densities of 10^4^ CFU per mL. For each of the 5 pairwise combinations, cocultures with six replicates were incubated in 6 mL of KB medium at 30°C and 180 rpm for 5 days. A volume of 20 μL of suspension from each flask was collected every 24 h for 5 days to determine the relative abundances of each strain via plating on KB agar plates. Additional two-species competition experiments were further performed in an identical dual-culture system using three different initial fractions of 1:1, 1:100, and 100:1, which were propagated through four growth-dilution cycles. For the 1:1 fraction, the inoculation was performed by mixing SBW25 and *Cupriavidus* sp. at an equal cell density (10^4^ CFU mL^−1^). For the 1:100 fraction, SBW25 was diluted 100-fold prior to mixing, and for the 100:1 fraction, *Cupriavidus* sp. was diluted 100-fold prior to mixing. For each growth dilution cycle, the dual cultures were serially diluted into fresh KB medium by a factor of 1:100. The population densities of SBW25 and *Cupriavidus* sp. were determined at the end of each incubation cycle.

To examine strain diversification in the presence of the competitor strain, we used the model of the adaptive radiation of SBW25 cultured in spatially structured static microcosms. Briefly, the *Cupriavidus* sp. and SBW25 were cocultured in 6 mL of KB medium containing 5% soil suspension using 25-mL flat-bottom glass bottles. The microcosms with six replicates were incubated without shaking to produce a spatially heterogeneous environment. The control treatment was included in the absence of a competitor strain. A total of 180 samples (6 treatments × 6 replicates × 5 times) were used to track taxon diversification at 28°C for 5 days. Destructive sampling of six replicates was performed every day. The diversity (Shannon index) was calculated by counting the morphology of colonies using spread plating on KB agar plates (66). To infer species interactions, we compared observed cell densities to the sum of monoculture cell densities of the constituent species (67, 68). A ratio value of 1 indicates additive growth, that is, no interaction; values of <1 indicate potential antagonistic interactions; and values of >1 indicate potential facilitation.

### UHPLC-MS analysis of extracellular metabolites.

To address question *ii*, we conducted untargeted ultra-high-performance liquid chromatography-mass spectrometry (UHPLC-MS) analysis of extracellular metabolic compounds to disentangle the potential mechanism of competitive suppression induced by SBW25 on *Cupriavidus* sp. strains. Monoculture (Cup1, Cup3, and SBW25) and coculture (Cup1-SBW25 and Cup3-SBW25) experiments were performed in 100 mL of KB medium at 28°C and 180 rpm. Taxa were inoculated at equal population densities of 10^4^ CFU mL^−1^. We used 100 mL of sterile KB medium as the control treatment. After 4 days, culture suspensions were collected and centrifuged at 10,000 rpm for 10 min, and the supernatants were sterilized using 0.22-μm membrane filters. The obtained samples were freeze-dried and suspended in precooled 80% methanol and 0.1% formic acid. Sample fractions of 1 mL were incubated on ice for 5 min and centrifuged at 10,000 rpm and 4°C for 15 min. The supernatants were diluted to a final concentration of 53% methanol. Final sample aliquots of 20 μL were used for LC-MS analysis.

### Statistical analysis.

Pearson correlation and one-way analysis of variance (ANOVA) were performed using SPSS 23.0. Correlation matrixes based on Spearman’s rank correlation coefficients between soil properties, bacterivorous nematode abundances, ALP-producing bacterial community, and soil enzymatic activities were visualized using the “corrplot” package in R (69). Principal-coordinate analysis was conducted to assess the effect of experimental factors based on Bray-Curtis distances of ALP-producing bacterial composition using the “capscale” function of the vegan package, and statistical analyses were performed using the “permutest” function (60). Analysis of similarities (ANOSIM) was further carried out to explore whether there was a significant difference in the ALP-producing bacterial populations between treatments with and without nematode addition.

To address question *iii*, random forest modeling was used to estimate the impacts of important predictors on ALP activity, including soil properties, nematodes, and the ALP-producing bacterial community. The increase in mean square error between observations and predictions was measured to assess the contribution of each predictor (70). The accuracy of the results was calculated for each tree and then averaged across 500 trees (71). The model was constructed using the randomForest package (72), and the significance of the corresponding model and contribution were measured using the A3 and rfPermute packages, respectively (73, 74). Structural equation modeling was applied to illustrate the direct and indirect influences of these factors on ALP activity. The model analysis was performed with maximum likelihood estimation using AMOS 20.0. The chi-square value, goodness-of-fit index, Akaike information criterion root, and mean square error of approximation were used to evaluate the model fitness (75).

### Data availability.

All sequences of the *phoD* gene obtained in this study were deposited in the Sequence Read Archive of the NCBI database under accession number PRJNA658735.
