# Supplementary figures and images for: Nematode Predation and Competitive Interactions Affect Microbe-Mediated Phosphorus Dynamics
Source: mBio. 2022 Apr 14;13(3):e03293-21. doi: 10.1128/mbio.03293-21 (PMC9239175; doi:10.1128/mbio.03293-21)

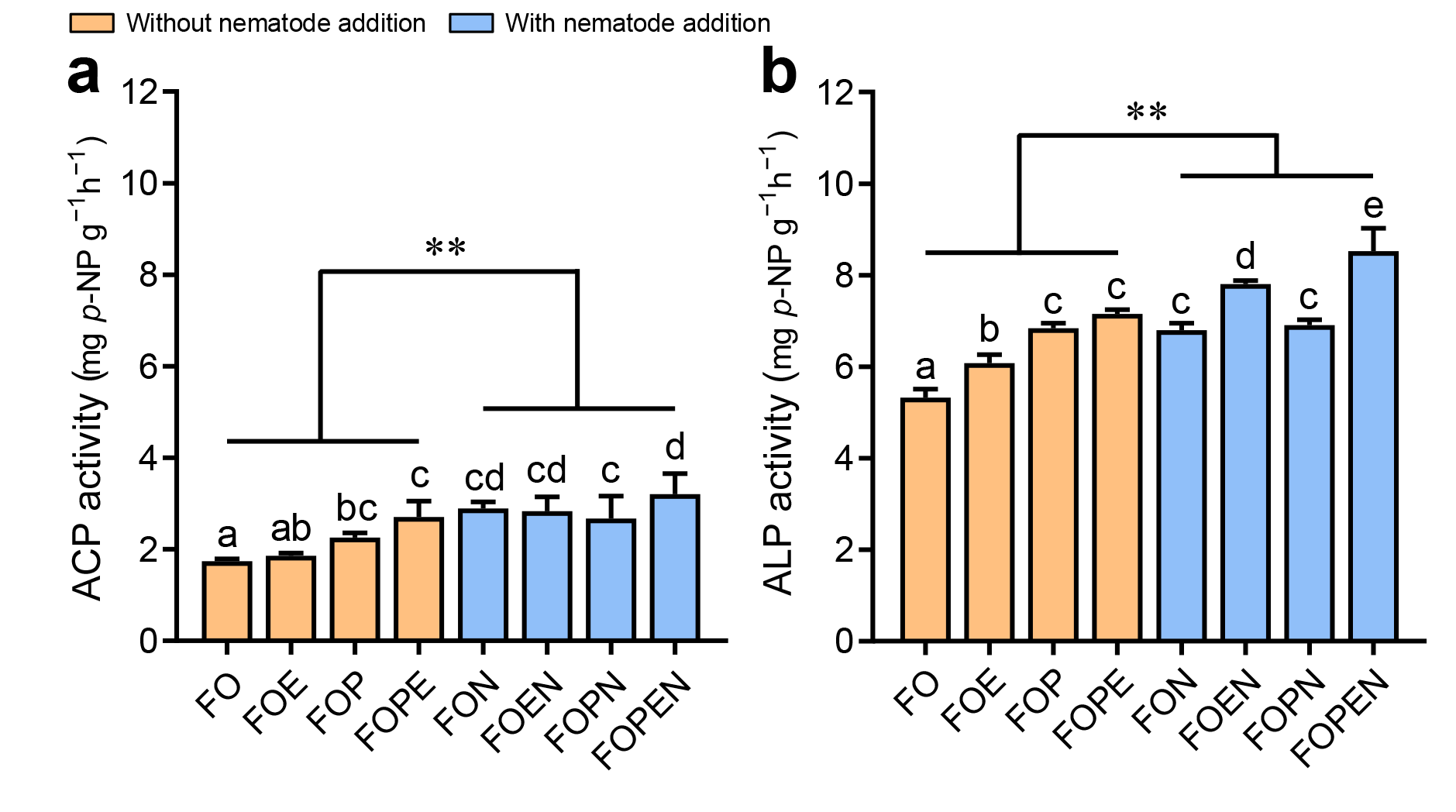

Supplement: FIG S1 [file mbio.03293-21-s0001.tif]

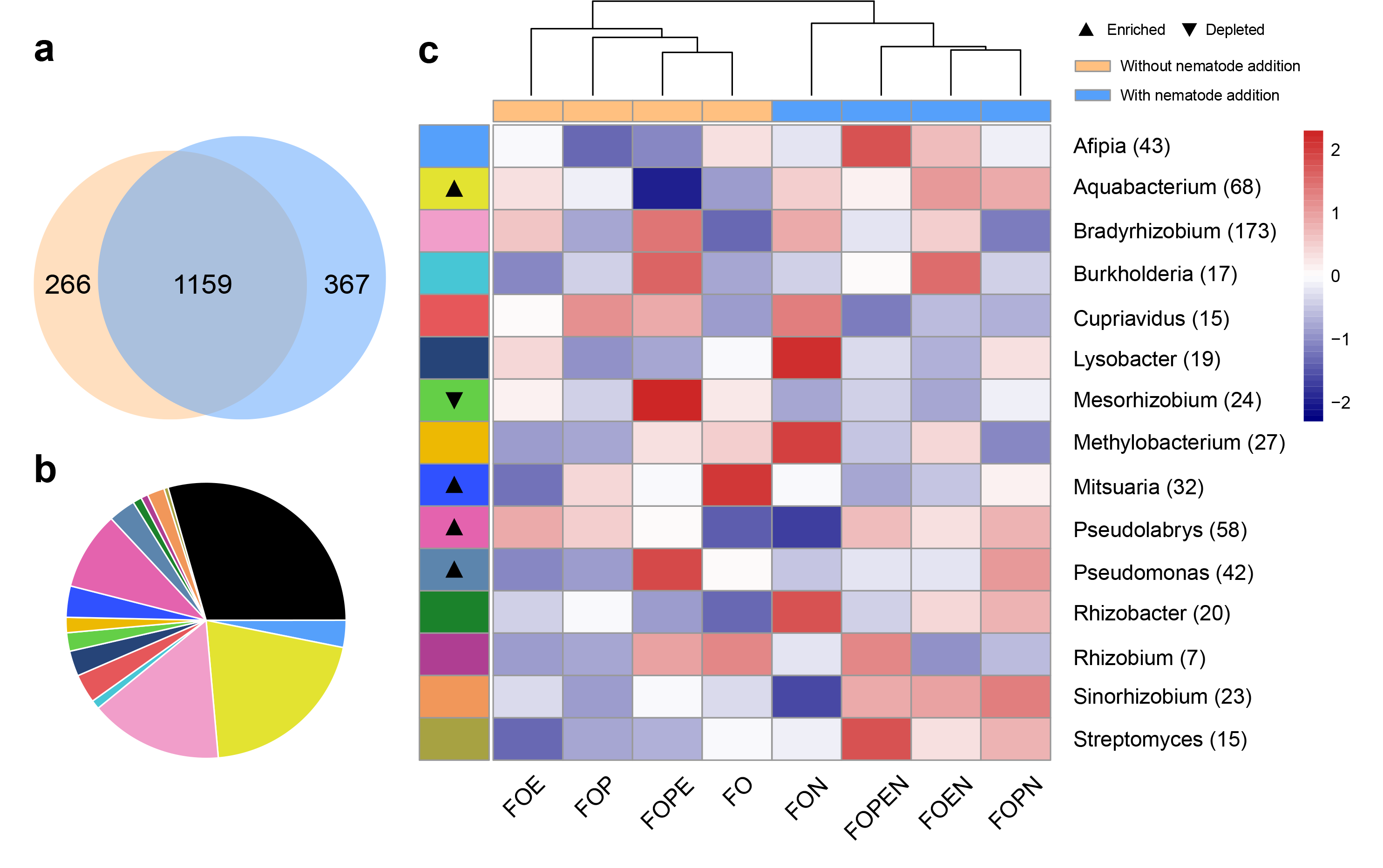

Supplement: FIG S2 [file mbio.03293-21-s0002.tif]

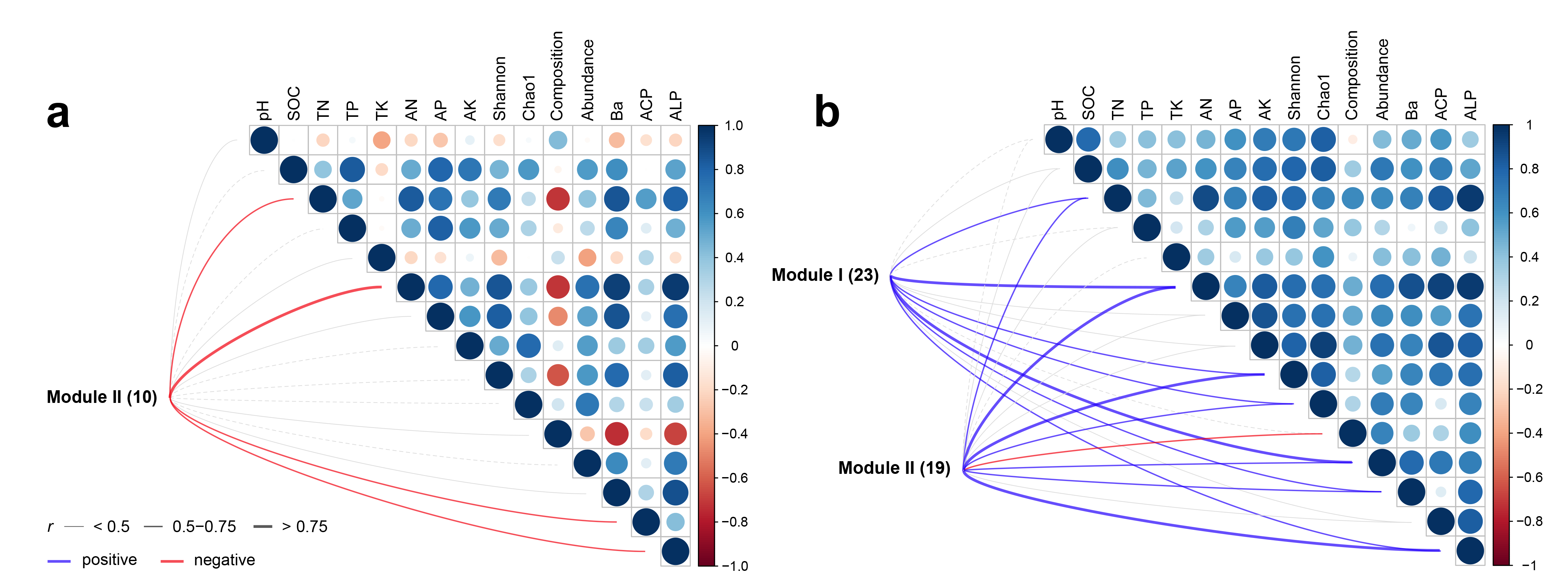

Supplement: FIG S3 [file mbio.03293-21-s0003.tif]

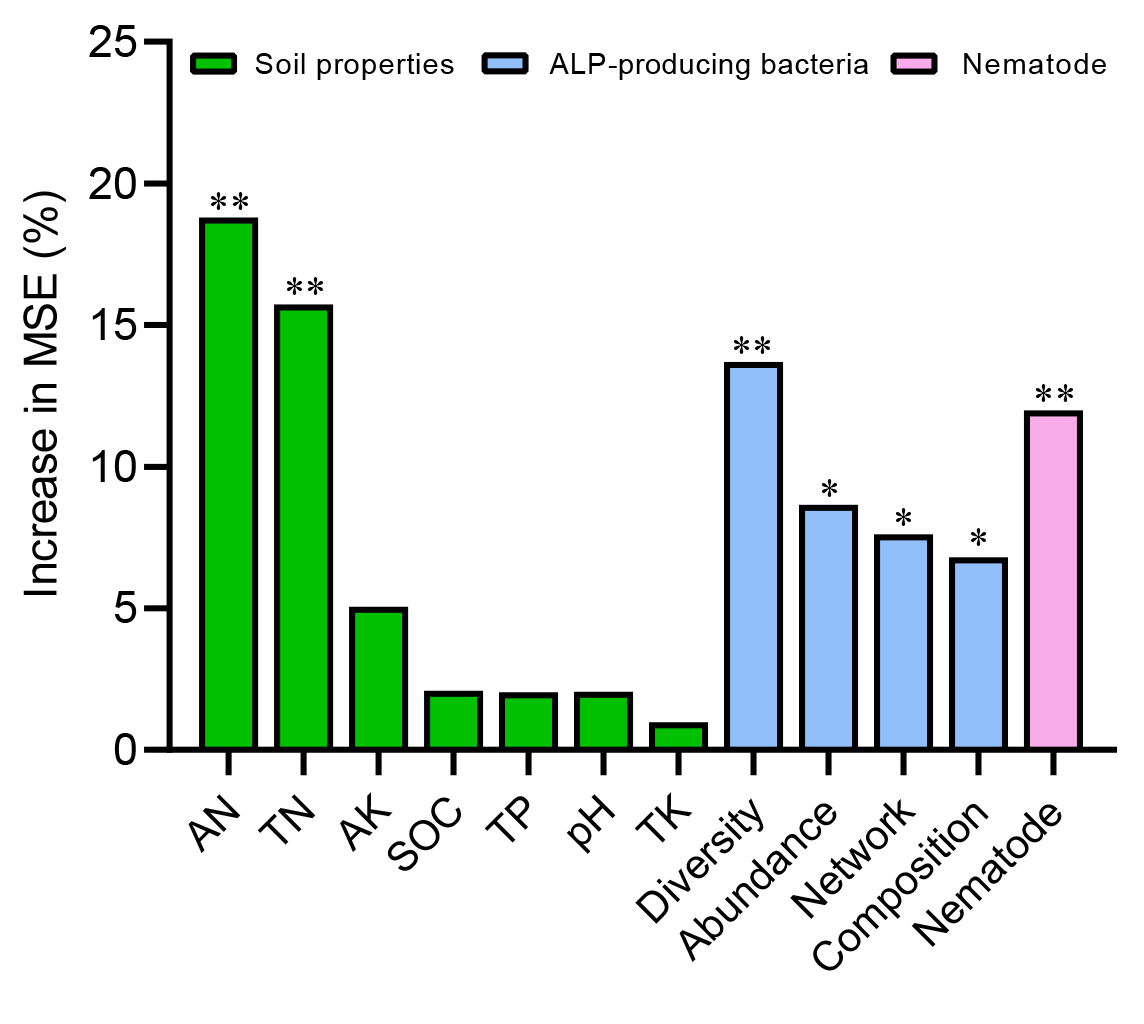

Supplement: FIG S4 [file mbio.03293-21-s0004.tif]

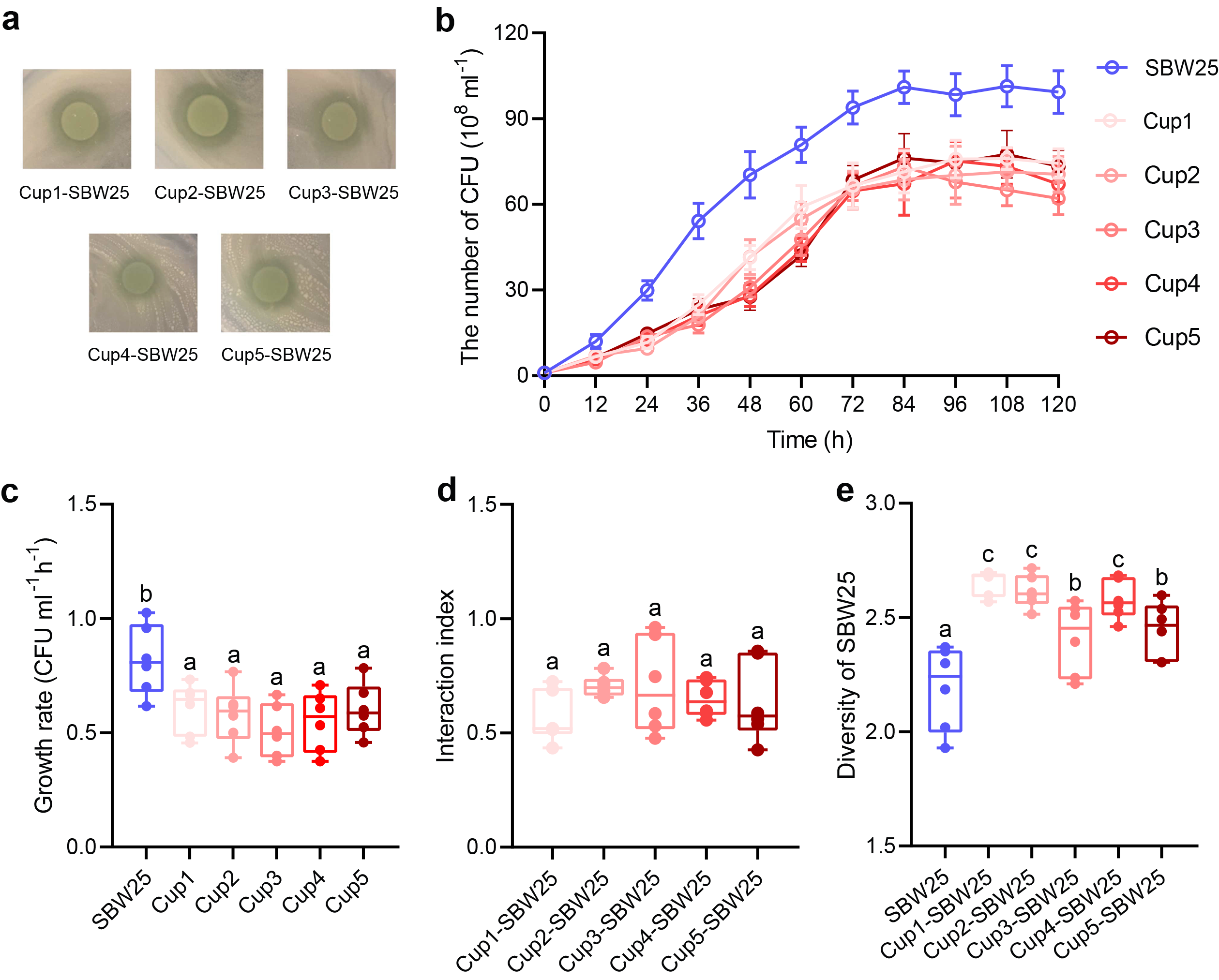

Supplement: FIG S5 [file mbio.03293-21-s0005.tif]
