# Supplementary material for: Nematode Predation and Competitive Interactions Affect Microbe-Mediated Phosphorus Dynamics
Source: mBio. 2022 Apr 14;13(3):e03293-21. doi: 10.1128/mbio.03293-21 (PMC9239175; doi:10.1128/mbio.03293-21)
Supplement: TABLE S1 [file mbio.03293-21-s0006.docx]

**Table S1** Rhizosphere Soil physicochemical properties under eight fertilization treatments^a^.

| Soil properties^b^ | FO | FOE | FOP | FOPE | FON | FOEN | FOPN | FOPEN |
| --- | --- | --- | --- | --- | --- | --- | --- | --- |
| pH | 6.31±0.17a | 6.21±0.13a | 6.24±0.04a | 6.4±0.16a | 6.38±0.26a | 6.21±0.16a | 6.43±0.22a | 6.17±0.3a |
| SOC (g/kg) | 7.98±0.12a | 8.28±0.25ab | 8.13±0.02ab | 8.49±0.35ab | 8.76±0.07bc | 8.36±0.29ab | 8.62±0.14bc | 8.89±0.12c |
| TN (g/kg) | 0.88±0.01a | 0.96±0.03b | 1.05±0.01b | 1.06±0.03b | 1.04±0.02b | 1.05±0.07b | 1.09±0.01bc | 1.15±0.02c |
| TP (g/kg) | 1.23±0.08a | 1.38±0.15ab | 1.47±0.1b | 1.25±0.06a | 1.46±0.02b | 1.45±0.02ab | 1.37±0.02ab | 1.45±0.02ab |
| TK (g/kg) | 12.32±0.1a | 12.58±0.97a | 11.85±0.59a | 13.25±0.94a | 12.87±0.35a | 12.49±0.43a | 12.53±0.34a | 12.23±0.57a |
| AN (mg/kg) | 74.18±1.57a | 79.31±1.37ab | 85.21±2.42b | 92.41±2.19c | 84.52±3.67b | 85.06±1.09b | 96.77±1.22cd | 105.75±3.2d |
| AP (mg/kg) | 92.98±3.47a | 101.95±1.72b | 103.72±0.43b | 106.11±1.64bc | 109.71±2.72bc | 109.28±2.19bc | 111.67±2.68c | 120.83±2.33d |
| AK (mg/kg) | 403.33±21.86a | 456.66±20.28ab | 496.66±17.63bc | 516.66±44.84bc | 523.23±23.33bc | 506.66±21.86bc | 523.33±28.48bc | 560.00±15.28c |

**a**. Values are the means (n=3) ± the standard error of the mean. Values in the same column followed by a lowercase letter indicate significant differences (*P* < 0.05) revealed by Tukey’s HSD test.

**b**. Soil properties include pH, soil organic carbon (SOC), total nitrogen (TN), total phosphorus (TP), total potassium (TK), alkali-hydrolyzale nitrogen (AN), available phosphorus (AP), and available potassium (AK).
