# Supplementary material for: Nematode Predation and Competitive Interactions Affect Microbe-Mediated Phosphorus Dynamics
Source: mBio. 2022 Apr 14;13(3):e03293-21. doi: 10.1128/mbio.03293-21 (PMC9239175; doi:10.1128/mbio.03293-21)
Supplement: TABLE S2 [file mbio.03293-21-s0007.docx]

**Table S2** Topological properties of ALP-producing bacterial co-occurrence networks between with (+N) and without (−N) nematode addition^a^.

| Topological properties | ALP-producing bacterial network | |
| --- | --- | --- |
|  | −N | +N |
| Number of nodes | 77 | 89 |
| Number of edges | 131 | 159 |
| Number of positive correlations | 104 | 107 |
| Number of negative correlations | 27 | 52 |
| Average path length (APL) | 4.628 | 4.725 |
| Graph Density | 0.044 | 0.049 |
| Network diameter | 12 | 13 |
| Average clustering coefficient | 0.239 | 0.246 |
| Average connectivity | 3.402 | 3.573 |
| Number of modules^b^ | 6 | 6 |
| Modularity (M) | 0.715 | 0.744 |

**a.** The numbers in parentheses indicate the nodes and edges observed in the stable isotope probing microcosms.

**b.** The number of modules with ≥5 nodes in networks.
